# Constitutive and Adaptive Traits of Environmental Stress Tolerance in the Threatened Halophyte *Limonium angustebracteatum* Erben (Plumbaginaceae)

**DOI:** 10.3390/plants11091137

**Published:** 2022-04-22

**Authors:** Ricardo Mir, Ignacio Romero, Sara González-Orenga, P. Pablo Ferrer-Gallego, Emilio Laguna, Monica Boscaiu, Lăcrămioara Oprică, Marius-Nicușor Grigore, Oscar Vicente

**Affiliations:** 1 Institute for the Conservation and Improvement of Valencian Agrodiversity (COMAV, UPV), Universitat Politècnica de València, Camino de Vera 14, 46022 Valencia, Spain; rimimo@upvnet.upv.es (R.M.); iromloz@posgrado.upv.es (I.R.); ovicente@upvnet.upv.es (O.V.); 2 Mediterranean Agroforestry Institute (IAM, UPV), Universitat Politècnica de València, Camino de Vera 14, 46022 Valencia, Spain; sagonor@doctor.upv.es (S.G.-O.); mobosnea@eaf.upv.es (M.B.); 3 Centre for Forestry Research and Experimentation (CIEF), CIEF-Wildlife Service, Generalitat Valenciana, Avda Comarques del País Valencia, 114, 46930 Quart de Poblet, Valencia, Spain; flora.cief@gva.es (P.P.F.-G.); laguna_emi@gva.es (E.L.); 4 Faculty of Biology, Alexandru Ioan Cuza University of Iasi, Bulevardul Carol I nr. 11, 700506 Iași, Romania; lacramioara.oprica@uaic.ro; 5 Faculty of Medicine and Biological Sciences, “Ștefan cel Mare” University of Suceava, Str. Universității 13, 720229 Suceava, Romania

**Keywords:** salt glands, recretohalophytes, endemism, water deficit, salt stress, ion transport, osmolytes accumulation, salt tolerance, drought tolerance, conservation programmes

## Abstract

*Limonium angustebracteatum* is a halophyte endemic to the E and SE Iberian Peninsula with interest in conservation. Salt glands represent an important adaptive trait in recretohalophytes like this and other *Limonium* species, as they allow the excretion of excess salts, reducing the concentration of toxic ions in foliar tissues. This study included the analysis of the salt gland structure, composed of 12 cells, 4 secretory and 8 accessory. Several anatomical, physiological and biochemical responses to stress were also analysed in adult plants subjected to one month of water stress, complete lack of irrigation, and salt stress, by watering with aqueous solutions of 200, 400, 600 and 800 mM NaCl. Plant growth was inhibited by the severe water deficit and, to a lesser extent, by high NaCl concentrations. A variation in the anatomical structure of the leaves was detected under conditions of salt and water stress; plants from the salt stress treatment showed salt glands sunken between epidermal cells, bordered by very large epidermal cells, whereas in those from the water stress treatment, the epidermal cells were heterogeneous in shape and size. In both, the palisade structure of the leaves was altered. Salt excretion is usually accompanied by the accumulation of salts in the foliar tissue. This was also found in *L. angustebracteatum*, in which the concentration of all ions analysed was higher in the leaves than in the roots. The increase of K^+^ in the roots of plants subjected to water stress was also remarkable. The multivariate analysis indicated differences in water and salt stress responses, such as the accumulation of Na and Cl, or proline, but K^+^ homeostasis played a relevant role in the mechanism of tolerance to both stressful conditions.

## 1. Introduction

The genus *Limonium* Miller (Plumbaginaceae) represents a fascinating halophytic model for understanding the functioning of coastal ecosystems. This genus is one of the most complex taxonomic groups in the Mediterranean flora [1]. Taxonomic research in the Iberian Peninsula revealed the existence of many endemics with narrow distribution ranges [1,2], including recently described new species from southeastern Spain (see, e.g., in the Valencian Community [3,4,5]).

The Valencian Community is one of the richest European territories in species of the *Limonium* genus [6]. *Limonium angustebracteatum* Erben is a species of sea lavender endemic to E and SE Spain (Alicante, Almeria, Castellón, Murcia and Valencia provinces) growing on argillaceous-sandy soils in littoral salt marsh halophytic communities. This species was described by Erben [7] from material collected in Sagunto, on “Playa del Puig”, a locality near Valencia City (Valencia province, Spain).

*Limonium angustebracteatum*, like other species of this genus, combines morpho-anatomical, biochemical and physiological traits that enable its growth in saline environments. *Limonium* species are known as recretohalophytes, as they have salt glands concentrated mainly in their leaves. This adaptation to saline environments occurs only in a few species of different families, including Plumbaginaceae [8]. Salt glands allow the removal of excess salts and play an essential role in regulating the internal ionic composition of leaves and ensuring osmotic balance, which, together with efficient osmotic adjustments, help prevent the dehydration of leaf cells [9]. Salt glands are reported in 87 species of *Limonium* [10], and they play an essential role in salt tolerance in this genus [11,12]. In addition to eliminating salts through glands, *Limonium* species accumulate toxic ions in their vacuoles, ensuring low-cost osmotic adjustment and avoiding ion toxicity, a common mechanism in dicotyledonous halophytes [13,14,15,16]. Under stress conditions, osmotic balance is also ensured by the synthesis and accumulation of osmolytes, or compatible solutes, in the cytoplasm. These are chemically diverse, the most common being proline and other amino acid derivatives, glycine betaine and other quaternary ammonium compounds, soluble sugars and polyols or sugar alcohols [17,18]. In addition to their primary function in osmotic adjustment [19], osmolytes also play many other roles, such as chemical chaperones, signalling molecules, modulators of gene expression or scavengers of “reactive oxygen species” (ROS) [17,18,19,20]. Quantifying the levels of ions and osmolytes in plants subjected to increased salt concentrations under controlled greenhouse conditions is of great relevance for understanding salt tolerance mechanisms in halophytes.

It is necessary to distinguish between constitutive stress tolerance mechanisms, present even in the absence of stress, and induced mechanisms, activated in response to stress. Constitutive mechanisms have a genetic basis and are species-specific; they include, for example, succulence or salt glands in some extremophiles [17] and other anatomical characteristics, such as those related to the reduction of water loss under high salinity conditions [21], or root structure [22]. However, halophytes, especially extremophiles, also possess other built-in mechanisms and are metabolically pre-adapted to salinity [23,24]. Studies on the halophyte *Eutrema salsugineum* revealed a phenotypic and metabolic adaptive plasticity not found in the related species *Arabidopsis* *thaliana* [25]. Many genes induced by salt stress in glycophytes are constitutively expressed at high levels in *E. salsugineum* [26]. The additional activation of induced stress responses at the transcriptional level occurs only at higher salinities, as reported in *E. salsugineum* compared to *Arabidopsis*, in agreement with the big difference in salt tolerance observed between these two species [27]. Metabolic pre-adaptation implies that extremophile species can show, even in the absence of salt, elevated levels of metabolites that are usually salt-induced; in addition, they can also respond to increased levels of salt stress by accumulating additional osmolytes not synthesised at lower salinities, as reported in different taxa, including *Limonium* species [28,29].

The aim of this study was two-fold. Firstly, we undertook a detailed study of the (yet unknown) morphology of the salt glands of *Limonium angustebracteatum* by Cryo-Field Emission Scanning Electron Microscopy (Cryo-FESEM) and toluidine blue-stained leaf sections. Secondly, we aimed to test the species’ tolerance to abiotic stress and elucidate its main tolerance mechanisms by analysing the effects of salinity and severe water stress under controlled experimental conditions on the plants’ anatomical structures, growth and biochemical parameters. These analyses included the determination of photosynthetic pigments, ions (Na^+^, Cl^−^, K^+^ and Ca^2+^) and osmolytes (proline, glycine betaine and total soluble sugars) contents and their changes in response to the applied stress treatments.

## 2. Results

### 2.1. Effects of Stress Treatments on Plant Growth

**Figure 1:**
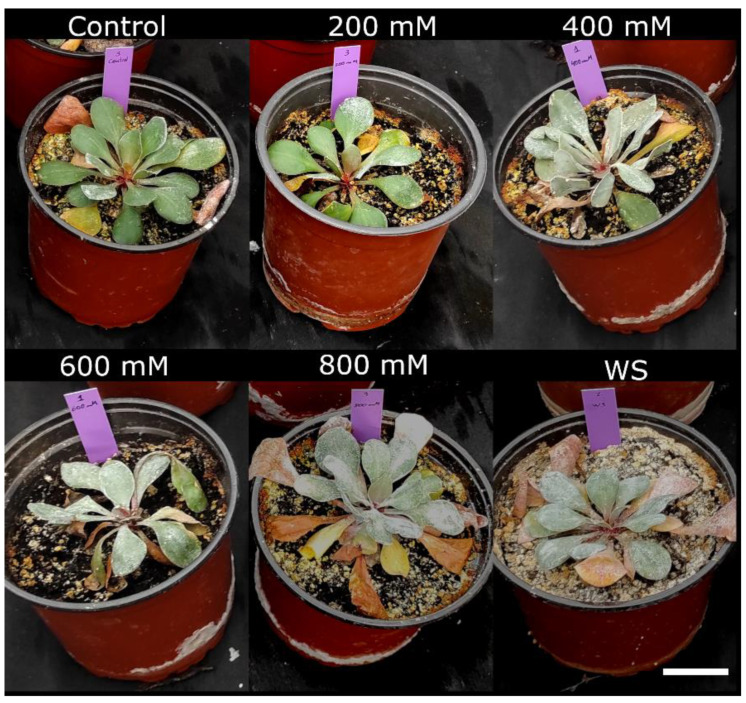
Effect of four weeks of salt treatments with the indicated NaCl concentrations, or water stress (WS), on *Limonium angustebracteatum* plants. The scale bar represents 3 cm.

One-year-old *Limonium* plants were subjected to different salt and water stress treatments as described in the Material and methods section. After four weeks of treatment, the effects of stress were visually observed in the overall growth of the plants. The strongest growth inhibition was registered in plants subjected to water stress, whereas those from the salt treatments did not show large variations with respect to the control non-stressed plants (Figure 1).

**Figure 2:**
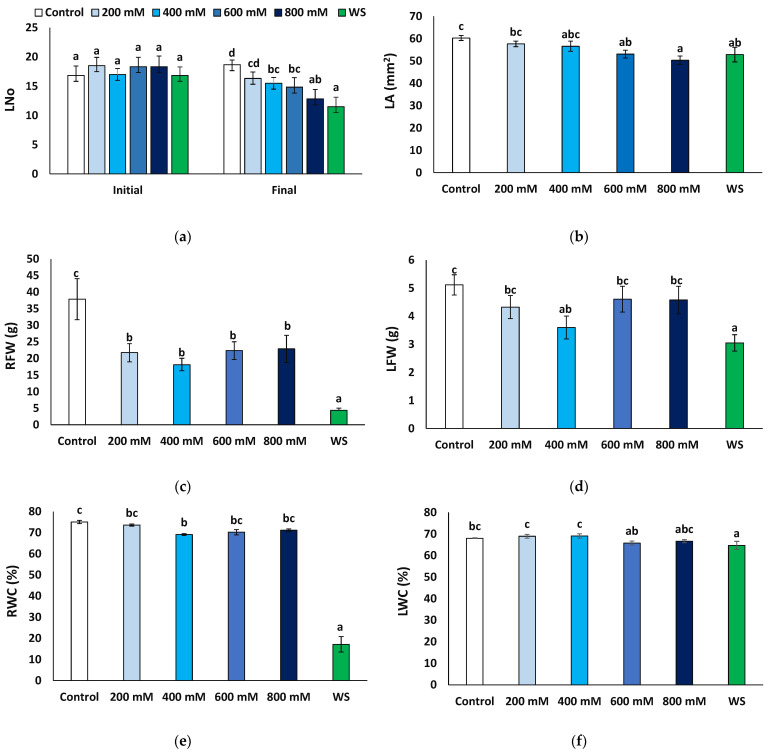
Growth parameters after four weeks of salt treatments with the indicated NaCl concentrations (X-axis), or one month of water stress (WS), of *Limonium angustebracteatum* plants. (**a**) Number of leaves, Lno, (**b**) leaf area, LA, (**c**), root fresh weight, RFW, (**d**), leaf fresh weight, LFW, (**e**) root water content, RWC, and (**f**) leaf water content, LWC. Means ± SE, n = 6. Different lowercase letters above the bars indicate significant differences between treatments, according to Tukey’s test (α = 0.05).

The number of leaves at the end of the treatments was compared to that at the beginning of the experiments. Except for the control plants, irrigated with non-saline water, where some new leaves were produced (1.8 per plant, on average), the number of leaves decreased in all other treatments, especially in the presence of 800 mM NaCl and under water stress conditions (Figure 2a). The mean leaf area also decreased compared to the control, significantly in plants of the 600 and 800 NaCl and the water stress treatments (Figure 2b). Root fresh weight decreased in all cases with respect to the control, notably in water-stressed plants, where an eight-fold reduction was recorded (4.34 vs. 37.87 g); although significant, the salt-induced reduction in root fresh weight was much less pronounced, down to about 50% of the control, without significant differences between the salt treatments (Figure 2c). Average values of leaf fresh weight were also lower in the stressed plants than in the non-stressed control, but the differences were significant only in the presence of 400 mM NaCl or after the water stress treatment (Figure 2d); in any case, this reduction (30−40% of the control) was not as substantial as for the root fresh weight. Another relevant parameter is the water content of the plants, calculated by the ratio between fresh and dry weight. As expected, water content decreased significantly in the water-stressed plants, especially in the roots (17.1% compared to 75.0% in control plants, Figure 2e) and to a much lower extent in the leaves (64.7% vs. 68.0%, Figure 2f). A significant but lesser reduction was also recorded in the roots of the 400 mM NaCl-treated plants (69.1%, Figure 2e) and in the leaves of the plants subjected to 600 mM NaCl (65.8%, Figure 2f).

### 2.2. Ultrastructure of Salt Glands

**Figure 3:**
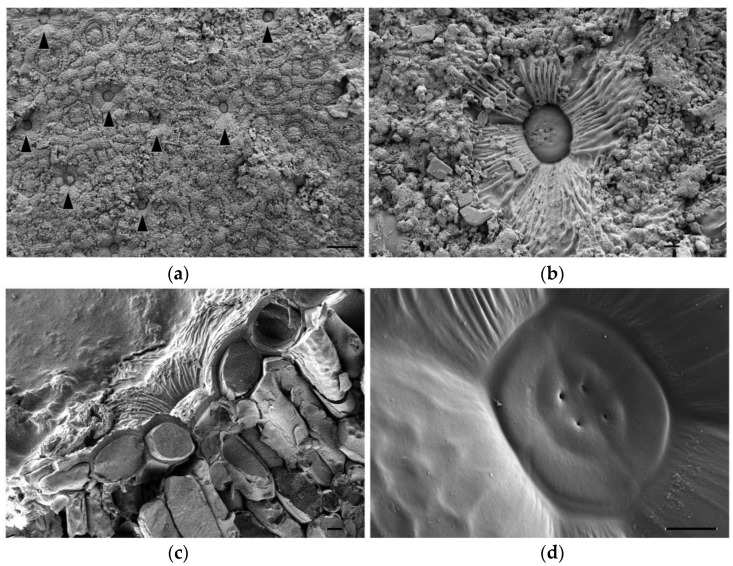
Cryo-FESEM images of salt glands of *Limonium angustebracteaum* grown under control conditions. (**a**) Salt glands (indicated by black arrowheads) located on the leaf epidermis. (**b**) Close look up of a salt gland on the leaf epidermis. Notice salt crystals’ excretion. (**c**) Transversal section of a salt gland located on leaf epidermis pits. (**d**) Detailed image of the salt gland core. Only two rings of salt gland cells are easily noticeable on this SEM image: 4 secretory cells in the centre, each of them with a secreting pore; 4 accessory cells surrounding the inner ring of secretory cells; just beneath it, a border of another accessory cell (bottom left) can be observed. Scale bar: 100 μm (**a**), or 10 μm (**b**–**d**).

Secretory salt glands are described in several genera belonging to the Plumbaginaceae family, including *Limonium* [19]. To better characterise these structures in *Limonium angustebracteatum*, we observed their anatomy using Cryo-FESEM microscopy. Salt glands were located at the epidermis level of leaves, and secreted salt was deposited on the leaf surface (Figure 3a,b). Based on our observations, we suggest that *L. angustebracteatum* salt glands are organised structures formed by 12 specialised cells (Figure 3c,d): four secretory cells containing a secretory pore that form an inner quadrant, an external ring of four evident accessory cells, and likely, just beneath, another quadrant of four accessory cells (Figure 3c,d).

### 2.3. Effects of Stress Treatments on Anatomical Structures

**Figure 4:**
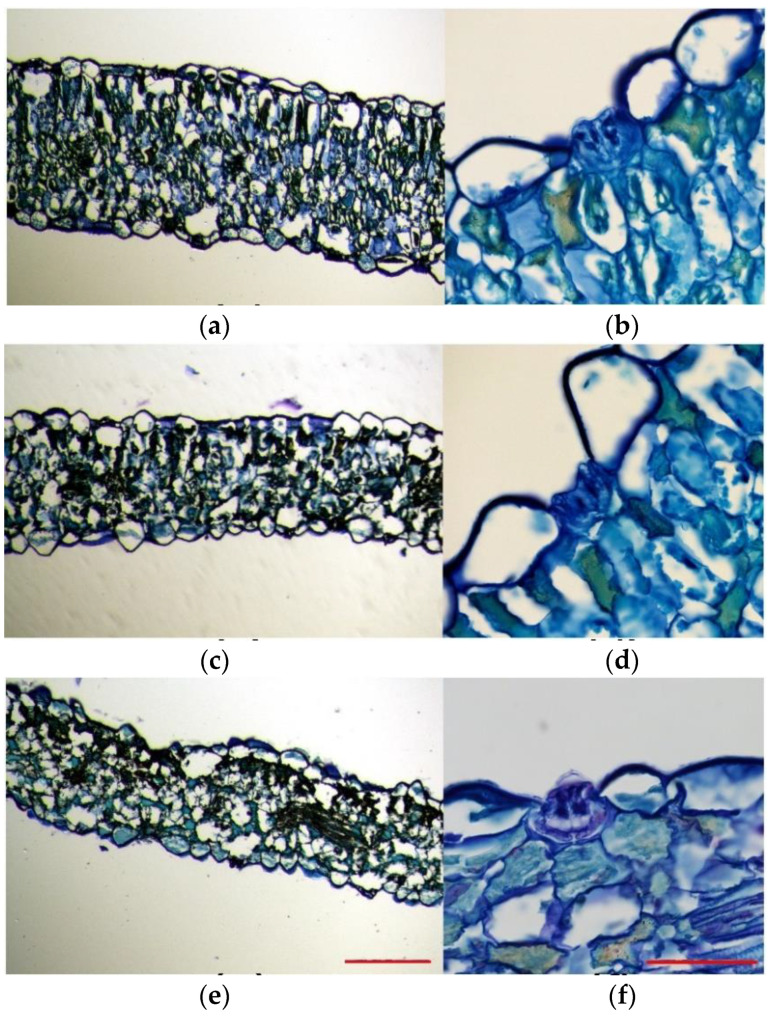
Toluidine blue staining of 8 mm leaf cross-sections of *Limonium angustebracteaum* plants under (**a**,**b**) control conditions, (**c**,**d**) after one-month 600 mM NaCl treatment and (**e**,**f**) after one-month water deficit treatment. Scale bar (**a**,**c**,**e**) 500 μm, and (**b**,**d**,**f**) 50 μm (n = 3).

Toluidine blue-stained leaf sections excised from plants subjected to salt and drought treatments were compared to those of control plants. This experiment allowed us to analyse the overall leaf anatomy, as well as the leaf cell size and morphology. In the control plants, leaf cross-sections were generally thicker than those of salt-treated and drought-stressed plants. Moreover, they were more compact and had a higher degree of tissue organisation (Figure 4a,c,e). The leaf structure of the plants grown under control conditions was bifacial dorsiventral, with palisade tissue towards the upper epidermis and spongy tissue beneath the lower epidermis. In these leaves, palisade tissue consisted of 1–3 layers of long cells, with regular disposition, without air spaces between them, being consequently more compact (Figure 4a). On the contrary, a typical palisade tissue was hardly noticeable in the plants subjected to salt stress, probably due to the lax leaf structure, and, although they presented 1–2 layers of palisade cells, they were short, with a slightly disorganised disposition, and presented air spaces between them (Figure 4c). In plants subjected to the drought treatment, as compared to the control plants, their epidermal leaf cells were heterogeneous in shape and size and ranged from very large and aculeiform-like shaped cells to large cells with no regular shape (Figure 4e). As in the case of the 600 mM NaCl salt treatment, at the analysed cross-section level, the leaf lost its bifacial structure so that the palisade tissue was no longer noticeable; instead, large irregularly shaped cells, with air spaces between them, occupied its position (Figure 4e).

Epidermal cells were generally homogeneous and mostly presented a thin and flattened morphology in plants grown under control conditions (Figure 4a). However, epidermal cells belonging to leaves from stressed plants presented heterogeneous shapes (Figure 4c,d), especially those that border salt glands, which were very large, and some showed an aculeiform appearance (Figure 4b,d,f). In all cases, stomata were noticeable on both the upper side and the underside of leaves; thus, the lamina was amphistomatic. Stomata appeared to be generally located at the level of epidermal cells and showed a very large substomatic chamber (Figure 4a,c,e). Salt glands were located in the upper epidermis in plants growing under all tested conditions and were sunken in the epidermis (Figure 4b,d,f). This was especially evident for salt-watered plants (Figure 4d). Generally, the epidermal cells surrounding the salt glands were larger in stressed plants than in plants watered with the control solution (Figure 4b,d,f).

### 2.4. Photosynthetic Pigments

**Figure 5:**
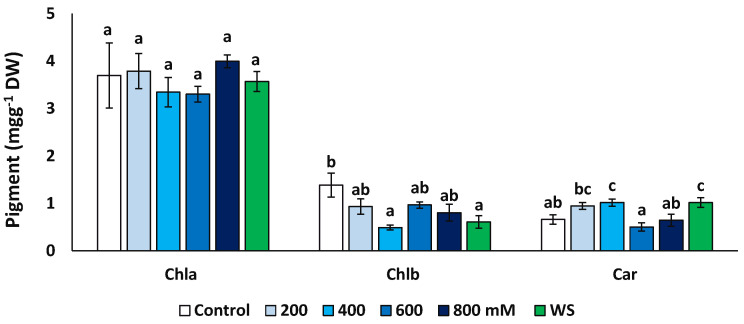
Variation of photosynthetic pigments contents in *Limonium angustebracteatum* leaves after one month of treatment with the indicated NaCl concentrations or one month of water stress (WS). Means ± SE, n = 6. For each pigment, different lowercase letters above the bars indicate significant differences between treatments, according to Tukey’s test (α = 0.05). Abbreviations: Chla, chlorophyll a; Chlb, chlorophyll b; Car, carotenoids.

The concentrations of chlorophyll a (Chl a), chlorophyll b (Chl b) and total carotenoids (Car) were measured at the end of the treatments in fresh leaf material (Figure 5). Concentrations of Chl a did not vary significantly in plants from different treatments, but Chl b decreased significantly, down to about 35% of the control in the presence of 400 mM NaCl (0.49 vs. 1.39 mg g^−1^ DW) and to ca. 44% for the water stress treatments (0.61 mg g^−1^ DW). An opposite variation pattern was observed for carotenoids, which showed significantly higher values in the presence of 400 mM NaCl and upon the water stress treatment than in the control.

### 2.5. Effects of Stress Treatments on Ion Contents

**Figure 6:**
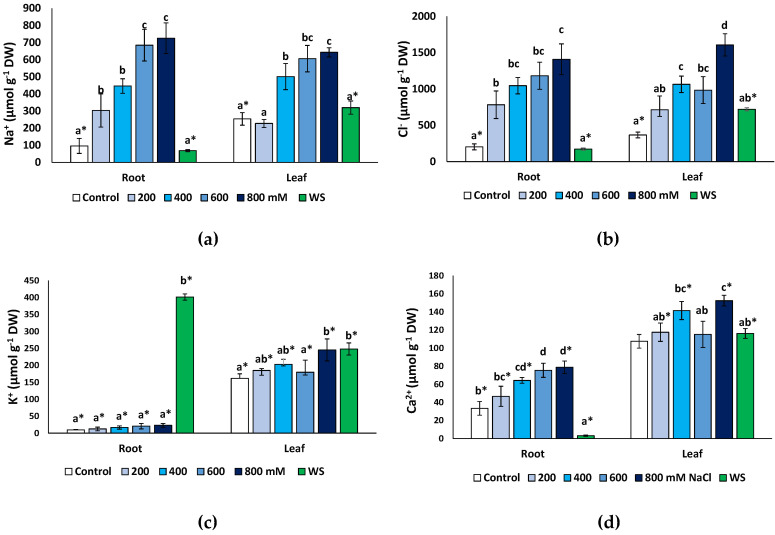
Ion contents in roots and leaves of *Limonium angustebracteatum* plants after one month of treatment with the indicated NaCl concentrations or one month of water stress (WS). (**a**) Na^+^, (**b**) Cl^−^, (**c**) K^+^, (**d**) Ca^2+^. Means ± SE, n = 6. For each organ, different lowercase letters above the bars indicate significant differences between treatments, according to Tukey’s test (α = 0.05). Asterisks indicate significant differences between ion concentration values in roots and leaves for the same treatment.

Monovalent ions (Na^+^, K^+^ and Cl^−^) and Ca^2+^ concentrations were measured at the end of the treatments in dry root and leaf material. As expected, both Na^+^ (Figure 6a) and Cl^−^ (Figure 6b) increased significantly under salt stress but not in water-stressed plants. A maximum Na^+^ concentration was found in the presence of 800 mM NaCl, reaching 7.5-fold higher values than in the non-stressed control in the roots (725 μmol g^−1^ DW, compared to 96 μmol g^−1^ DW) and a 2.5-fold increase (642 vs. 254 μmol g^−1^ DW) in the leaves (Figure 6a). A similar pattern of variation was established for Cl^−^ (Figure 6b), with maximum measured concentrations of 1408 μmol g^−1^ DW in the roots and 1606 μmol g^−1^ DW in leaves of the 800 mM NaCl-treated plants; these values represent relative increases over the corresponding controls of, approximately, 7-fold and 4.4-fold in roots and leaves, respectively. In general, the Na^+^ and Cl^−^ concentrations in the salt-treated plants were the same in roots and leaves at all external salinities tested. However, interestingly, in non-stressed controls and plants subjected to water stress, the contents of both ions were significantly higher in the leaves than in the roots (Figure 6a,b).

An unusual pattern of variation was found for K^+^ in roots (Figure 6c). Average contents of this cation increased in roots in response to salt stress, up to three-fold higher than in the control in the presence of 800 mM NaCl (ca. 27 vs. 9 μmol g^−1^ DW), but these differences were not statistically significant. However, water deficit stress caused a considerable increase in K^+^ concentration to more than 380 μmol g^−1^ DW, or 42-fold over the control values. In the leaves (Figure 6c), a modest (ca. 1.5-fold) but significant increase in K^+^ was detected in response to the highest salt concentration tested (800 mM NaCl) and to water stress. Leaf K^+^ levels were significantly higher than those in the roots, in control plants and at all NaCl concentrations; only in water-stressed plants, the opposite correlation was observed, with K^+^ contents substantially higher in the roots than in the leaves (Figure 6c).

Finally, Ca^2+^ concentrations showed patterns of variation roughly similar, qualitatively, to those of Na^+^ and Cl^−^, with significant increases over control values, in roots and leaves, in the presence of 400 mM NaCl and higher salt concentrations (Figure 6e). Leaf Ca^2+^ contents did not vary significantly in response to water deficit but decreased sharply, about 10-fold, in the roots of water-stressed plants (Figure 6e). Ca^2+^ concentrations were significantly higher in the leaves than in the roots in the controls, in all salt treatments and, especially, in the water-stressed plants (Figure 6e).

### 2.6. Effects of Stress Treatments on Osmolyte Accumulation

Proline (Pro), glycine betaine (GB) and total soluble sugars (TSS) represent the most common plant osmolytes, which are synthesised and accumulate in the cells contributing to osmotic adjustment under abiotic stress conditions causing cell dehydration. All three were analysed in fresh leaf material from plants sampled at the end of the experiment after one month of exposure to stress.

**Figure 7:**
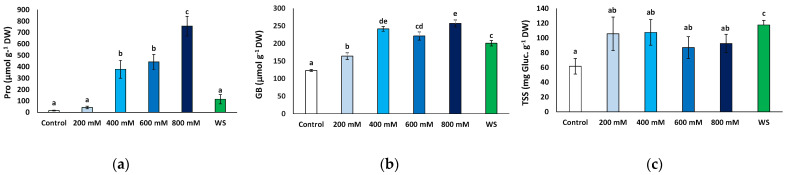
Osmolyte contents in leaves of *Limonium angustebracteatum* plants after one month of treatment with the indicated NaCl concentrations or one month of water stress (WS). (**a**) proline (Pro), (**b**) glycine betaine (GB), (**c**) total soluble sugars (TSS). Means ± SE, n = 6. Different lowercase letters above the bars indicate significant differences between treatments, according to Tukey’s test (α = 0.05).

Pro contents increased significantly in response to salt stress, at 400 mM and higher NaCl concentrations, from 15.7 to 756.6 μmol g^−1^ DW, in the control and 800 mM NaCl-treated plants, respectively, representing a 48-fold increase. Conversely, leaf Pro contents in plants subjected to the water deficit treatment did not differ significantly from those measured in control plants or at moderate (200 mM NaCl) salinity (Figure 7a).

GB contents showed a significant increase in response to water deficit and all salt stress treatments with respect to the control. However, the variation was not as marked as that of Pro, reaching no more than twice the control value (Figure 7b). It should be mentioned that this relatively small stress-induced increment in GB levels was primarily due to the presence of the osmolyte at high concentrations in non-stressed plants, ca. 123 μmol g^−1^ DW; that is, almost 10-fold higher than Pro background contents (Figure 7b).

The mean TSS leaf concentrations also increased in the stressed plants relative to the control, but this variation was only significant in the plants subjected to water stress, reaching almost twice the values measured in the control plants (117.7 vs. 61.6 mg eq. gluc g^−1^ DW) (Figure 7c).

### 2.7. Multivariate Analysis

**Figure 8:**
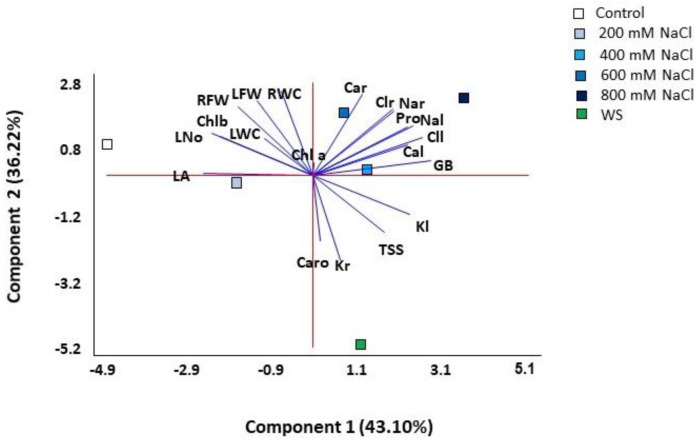
Biplot of the principal component analysis (PCA) conducted with the analysed traits in *Limonium angustebracteatum* plants subjected to one-month stress treatments. Abbreviations: LNo, number of leaves; LA, leaf area; RFW, root fresh weight; LFW, leaf fresh weight; RWC, root water content; LWC, leaf water content; Chla, chlorophyll a; Chl b, chlorophyll b; Caro, carotenoids; Na^+^_r,_ sodium in roots; Na^+^_l_ sodium in leaves; Cl^−^_r_, chlorine in roots; Cl^−^_l_, chlorine in leaves; K^+^_r_, potassium in roots; K^+^_l_, potassium in leaves; Ca^2+^_r_, calcium in roots; Ca^2+^_l_, calcium in leaves; Pro, proline; GB, glycine betaine; TSS, total soluble sugars.

A principal component analysis (PCA) was performed on all quantitative traits analysed. Four components were detected with eigenvalues greater than one, covering 98.04% of the total variability of the data (Table 1, Figure 8). The first component explained 43.10% of the total variation and correlated (values greater than 0.25, shown in bold font in Table 1) positively with the concentration of the osmolytes glycine betaine (GB) and proline (Pro) and with leaf concentrations of Na^+^, K^+^, Cl^−^ and Ca^2+^, and negatively with the leaf area (LA), leaf number (L No) and chlorophyll b (Chl b). The second component, which explained an additional 36.2% of the variation, was positively correlated (values above 0.25) with growth parameters (leaf and root fresh weight and root water content) and with root Na^+^, Cl^−^ and Ca^2+^ concentrations, and negatively with root K^+^ and carotenoids (Caro) levels. The first component separated the scores of control and salt-stressed plants, except for those of the 800 mM NaCl treatment, which were separated at one end of the second component, while those of water stress at the other end of the same axis (Figure 8). Thus, the most separated treatments on the two axes of the biplot were the control, water stress and 800 mM NaCl.

**Figure 9:**
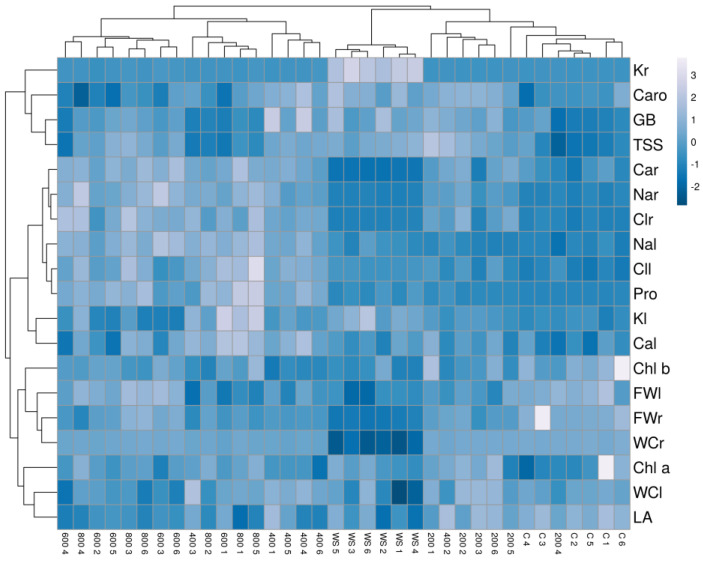
Hierarchical cluster analysis and heatmap of physiological and biochemical parameters in *Limonium angustebracteatum* plants subjected to one-month stress treatments. Abbreviations: LNo, number of leaves; LA, leaf area; RFW, root fresh weight; LFW, leaf fresh weight; RWC, root water content; LWC, leaf water content; Chla, chlorophyll a; Chl b, chlorophyll b; Caro, carotenoids; Na^+^_r,_ sodium in roots; Na^+^_l_ sodium in leaves; Cl^−^_r_, chlorine in roots; Cl^−^_l_, chlorine in leaves; K^+^_r_, potassium in roots; K^+^_l_, potassium in leaves; Ca^2+^_r_, calcium in roots; Ca^2+^_l_, calcium in leaves; Pro, proline; GB, glycine betaine; TSS, total soluble sugars.

The hierarchical cluster analysis (HCA) performed together with the heatmap (Figure 9) with traits measured in all plants confirmed the PCA results and revealed a clear separation of the drought-stressed plants in one cluster. In addition, the cluster topology allowed the separation of two other groups, one including plants from the control and the 200 mM NaCl treatments and the other from the 600 and 800 mM NaCl treatments. Plants treated with the intermediate salt concentration (400 mM NaCl) showed a heterogeneous pattern, with individuals falling into different clusters. Plants from the water stress treatment showed high positive correlations with root K^+^ contents and negative with root fresh weight, water content and Ca^2+^ concentration. The cluster of plants from the high salt treatments (600 and 800 mM NaCl) had positive correlations with root Na^+^ and Cl^−^ and negative with leaf area, leaf water content and carotenoid levels. Finally, plants from the control and the lowest salt concentration (200 mM NaCl) were positively correlated with growth parameters and negatively with ions and osmolytes contents (Figure 9).

## 3. Discussion

*Limonium angustebracteatum* is an endemic species of the Iberian Peninsula with a high conservation interest. Although it is still largely distributed in E and SE Spain, some populations declined significantly during recent decades. In the Valencian region, it is quoted as NT (Near threatened) [30] for the IUCN Red List Categories [31]. In addition, it is a key species for some protected habitats in this region, such as *Limonietalia* salt steppes, becoming particularly dominant in the rare plant association *Artemisio gallicae-Limonietum angustebracteati* Costa and Boira 1981 (see [32,33]).

Like other species of this genus, *L. angustebracteatum* is a recretohalophyte with salt excretion capacity through salt glands located in the aerial parts of the plants, which provide a relevant constitutive mechanism contributing to salt tolerance in these species. Salt glands are epidermal structures specialised in the storage and exclusion of salts present in halophytes of different families; they function by regulating the ionic balance and ensuring a stable osmotic pressure [26,34,35]. The great phylogenetic diversity of recretohalophytes, and their structural differences, suggest that this trait originated independently multiple times during plant evolution [36] and represents a clear example of convergent adaptation [37]. Salt glands were already described in classical plant anatomy studies [38], but recent research sheds light on their evolutionary origin and the physiological, biochemical and molecular mechanisms of salt secretion through salt glands [19,20,34,36,39]. There are many reports on the structure and function of salt glands in the genus *Limonium* [34,40,41,42,43,44] due to its large number of species, many of them endemics, adapted to saline or dry environments.

The ultrastructure of salt glands in Plumbaginaceae is problematic because of the large diversity of structural architectures reported for different genera and species and also the lack of a consistent and standardised language to define different types of component salt gland cells [19,38]. Furthermore, there are technical difficulties in handling anatomical cross-sections and electron microscopy images, which, in fact, can never reveal the entire structure and topography of different types of cells and their connections within a salt gland. For this reason, salt glands structures based on 12, 16 or 20 cells have been proposed, with different relative positions with respect to the epidermis level (summarised in [19]). Based on our analysis of *L. angustebracteatum*, we suggest a salt gland structure composed of 12 cells, 4 secretory cells forming an inner quadrant, an external ring of 4 completely visible accessory cells and just beneath it, another quadrant of 4 accessory cells. A similar 12-cells-based salt gland structure was also reported for other *Limonium* species: *L. aureum* (L.) Hill [45], *L. bellidifolium* (Gouan) Dumort. [46], *L. bocconei* (Lojac.) Litard., *L. pignanttii* Brullo and Di Martino or *L. lojaconoi* Brullo [47].

The number of salt glands is shown to increase with various treatments, such as Ca^2+^ [48], exogenous nitric oxide [49] or melatonin [41]; however, NaCl appears to be the main trigger for salt gland development [34,40,44]. Salt gland density is also correlated with the plants’ natural environment; for example, *Limonium* species growing under high salinity conditions have a higher density of glands than those present in less saline habitats [43].

The available data on the anatomical modifications of halophytes under salt and water stress are surprisingly scarce compared to glycophytes. In *L. angustebracteatum*, the large epidermal cells of salt and drought-exposed plants can be regarded in terms of increasing the thickness of, especially, the upper epidermal layer, as it is also reported for plants of the halophyte *Salvadora persica* subjected to high salinity levels [50]. In this species, leaf palisade tissue disappeared in the presence of 750 mM NaCl [50]; the same effect was observed in our experiments with *L. angustebracteatum* in salt-treated plants and, particularly, in plants exposed to drought. This disorganisation of typical palisade tissue might be an adaptation to minimise the photosynthetic energy utilisation under intense stress and could be correlated with the reduction of photosynthetic pigment levels in stressed plants. A palisade tissue with shorter cells was also reported in the halophyte *Juncus acutus* L., in the presence of 400 mM NaCl [51].

Regarding the physiological responses of *L. angustebracteatum*, high salt concentrations and water stress affected plant growth. All plants, including halophytes, respond to severe stress, reducing or even completely stopping their growth, as metabolic precursors and energy resources are used under such conditions to activate defence mechanisms rather than for biomass accumulation [52]. All glycophytes and most halophytes show optimum growth in the absence of salt. Only some extremophiles grow better in the presence of low or moderate salinity; even in these plants, high salt concentrations have inhibitory effects on growth [53]. The growth of halophytes of the genus *Limonium* is generally unaffected by low NaCl concentrations [54] or is, sometimes, even stimulated [55,56,57,58]. However, growth inhibition is observed at high salinities, 300 or 400 mM NaCl, even in the most salt-tolerant species [59,60]. Nevertheless, there are also *Limonium* species that are not halophytes but rather susceptible to even low concentrations of NaCl, such as *L. perezii* (Stapf) F.T. Hubb. and *L. sinuatum* (L.) Mill. [61] or *L. dufourii* [62,63]. *Limonium angustebracteatum* had optimal growth in the absence of salt, but no significant inhibition was observed in the presence of 200 M NaCl. The same response was found in other *Limonium* endemics of this geographic area [64,65]. This behaviour enables the species to grow in saline depressions but also in dunes and other elevated, permeable substrates, where soil salinity may be lower due to the leaching of ions.

Of all the tested treatments, the most significant growth inhibition was recorded in the plants subjected to water deficit by complete withholding of irrigation for one month; the effect of water stress was most notably reflected in a drastic reduction in root fresh weight and water content. This response, reported even for *Limonium* species growing in markedly dry areas [66], is explained by the severity of water stress in potted plants, where roots cannot extend in search of more profound and wetter soil layers as they do in their natural habitats [65]. Leaf senescence was accentuated by stress, as revealed by the loss of more than five leaves, on average, in the presence of 800 mM NaCl and the water deficit treatment. The remaining leaves had a smaller surface area, but photosynthetic pigment contents did not decrease substantially; only Chl b showed a significant, although slight, reduction under stress and carotenoid levels even increased, again slightly but significantly, in response to some stress treatments. These results indicate that *L. angustebracteatum* behaves as a typical perennial halophyte, tolerating high salt concentrations far beyond those found in the soil in its natural habitat [63]. Moreover, under controlled greenhouse conditions, it is more susceptible to severe water stress than moderate salinity, even though the plants survived the strong water deficit conditions applied. In their natural habitat, plants of this perennial species also survive the intense summer drought characteristic of the Mediterranean region, where periods of one to more than two months of absolute lack of precipitations are not uncommon.

Like all recretohalophytes, *Limonium* species secrete ions, especially Na^+^ and Cl^−^, through their glands [39,55,67]. However, these plants also can accumulate these ions in the cells, sequestering them in the vacuoles to avoid their toxic effects in the cytosol; inorganic ions represent a ‘cheap’ osmoticum used by many halophilic dicots for osmotic adjustment under stress [22,68]. Similar patterns of a concentration-dependent increase of Na^+^ and Cl^−^ in response to the NaCl treatments were found for the two monovalent ions and the two organs, roots and leaves. Interestingly, in non-stressed plants and under water deficit conditions, the concentrations of both ions were significantly higher in the leaves than in the roots; this difference was much more pronounced in the case of water-stressed plants. These data indicate that, even at low external salinity, these ions are actively transported from roots to leaves, where they are used for osmotic balance, as reported in other *Limonium* species [62,64,69,70].

An increase in Na^+^ is usually accompanied by a decrease in K^+^, as both cations compete for the same binding sites. Moreover, it is well known that excess Na^+^ causes the depolarisation of the plasma membrane, inducing the activation of outward rectifier K^+^ channels and thus the loss of cellular K^+^ [71,72]. K^+^ is an essential nutrient for plants, involved in many cellular and metabolic processes, such as cell elongation, stability of membrane integrity, enzyme activation, protein synthesis, photosynthesis, stomatal movement or phloem transport [73]. In stress resistance, the role of K^+^ is related to osmotic adjustment [74]. In *Limonium*, the activation of K^+^ transport from the roots to the aboveground organs was reported [62], which ensures that its leaf concentration is maintained or decreases only slightly with increasing salinity, thus counteracting, at least partly, Na^+^ deleterious effects. The same mechanism was described in other halophytes, such as *Plantago crassifolia* [60] or *Inula crithmoides* [75]. Contrary to the general behaviour, even salt-induced increases of K^+^ contents, relative to the control, were reported, for example, in the roots of several Mediterranean *Limonium* species [64] or the leaves of *L. stocksii* under low salinity [70]. Similar responses were observed in *L. angustebracteatum* plants subjected to salt treatments, where leaf K^+^ levels increased slightly with increasing salinity and were significantly higher than in roots at each NaCl concentration tested, including the non-stressed controls. This indicates that K^+^ uptake and transport to the leaves is an essential mechanism of salt tolerance in this species, contributing to osmotic balance, also in the absence of salt. Indeed, in situ subcellular localisation studies revealed that K^+^ accumulated in both the cytoplasm and the nucleus of salt gland cells under saline conditions, which may play an important role in salt secretion [76]. Leaf K^+^ contents under water deficit conditions were similar to those measured at the highest salinity, 800 mM NaCl, suggesting the participation of this cation in the responses of the plants to drought. However, an unusual pattern was found for the K^+^ concentration in the roots of *L. angustebracteatum* water-stressed plants, which increased more than 40-fold over the control values. The plants seem to use K^+^ uptake as a defence mechanism against drought, which strongly affects the root system. K^+^ increases in roots (although not so pronounced) and leaves were also reported in other congeners such as *L. girardianum* and *L. narbonense* [66]. Higher K^+^ content in leaves than in roots was also reported in plants of this genus sampled in natural environments [65].

Calcium is another essential cation involved in membrane and cell wall stabilisation, the regulation of ion transport and selectivity and enzymatic activities [77]. Under salt stress conditions, the external application of Ca^2+^ reduces the toxic effects of NaCl, presumably by facilitating increased K^+^/Na^+^ selectivity [77]. Ca^2+^ plays an essential role in stress signalling; cytosolic calcium activates the calcium sensor protein SOS3, which triggers a signalling cascade activating the plasma membrane Na^+^/H^+^ antiporter, SOS1, leading to Na^+^ efflux out of the cytosol to the apoplast and also contributing to Na^+^ compartmentalisation in the vacuole through the equivalent tonoplast antiporter, NHX1 [78,79]. Ca^2+^ levels increased in both roots and leaves in several species of this genus [64,70]; in other species, on the contrary, this cation was found to decrease with salinity [64,80]. The strong reduction of Ca^2+^ in the roots of water-stressed plants could be related to the high input of K^+^ ions, as the presence of other cations is known to profoundly influence Ca^2+^ uptake, with high levels of K^+^ and Mg^2+^ reducing Ca^2+^ uptake [81].

A fundamental mechanism to ensure osmotic balance and compensate for the accumulation of toxic ions in the vacuoles, mostly in dicotyledonous halophytes, is the synthesis of osmolytes [23]. The genus *Limonium* is notable for the variety of compatible solutes used concomitantly, even in the same species [54]. Proline, one of the most common plant osmolytes, is found in all *Limonium* species and can be considered a functionally relevant osmolyte in this genus [54]. Many reports in *Limonium* species indicate an increase in Pro contents under stress, and often higher Pro levels are correlated with higher stress tolerance [63]. However, this cannot be generalised to all *Limonium* taxa since, for example, in *L. latifolium*, the variation in Pro concentrations was considered to be related to damage and successive repair in the mitochondrial step of proline oxidation [82] rather than to its salt tolerance. In *L. angustebracteatum*, a linear increase of Pro contents in parallel to increasing salinity was observed, reaching over 750 μmol g^−1^ DW in the presence of 800 mM NaCl, but not in response to water stress, suggesting that this osmolyte plays a relevant role in the plant adaptation to salinity, but not so much to drought.

Glycine betaine is another common osmolyte in plants, present at high concentrations in the salt-tolerant Amaranthaceae and Poaceae [83] and acting as an osmoregulator under abiotic stress conditions [84]. GB is synthesised primarily from choline, and GB accumulators have particular adaptations in the biogenesis of choline and the methyl group that are not present in other plants [85], allowing accumulations of 4–40 µmol g^−1^ FW in spinach and sugar beet [84], or up to 900 µmol g^−1^ DW in the halophyte *Suaeda fruticosa* [86]. The values recorded in *L. angustebracteatum* (even in control plants) are above those previously reported in *Limonium* species sampled in natural environments [65,87,88]. Since GB concentrations increased significantly under both types of stress, we can conclude that this compatible solute plays a relevant role in the osmotic adjustment of *L. angustebracteatum.*

On the other hand, soluble sugars do not seem to participate in the salt or drought tolerance mechanisms in *L. angustebracteatum*. TSS levels did not vary significantly in plants subjected to the salt treatments, and the increase observed in response to water deficit was too small to have any relevant osmotic effect. Nevertheless, it is always difficult to assess the possible role in stress tolerance mechanisms of changes in sugar concentrations because of their multiple biological functions not directly related to stress responses, as direct products of photosynthesis, metabolic precursors or energy sources [89].

These results suggest that *Limonium angustebracteatum* can behave as a eurioic species, particularly regarding the soil salt concentration. This would imply that the species can easily adapt to environmental changes, even though it has little resistance to prolonged periods of flooding, according to our observations in the field (EL and PFG, pers. obs.). In this respect, *L. angustebracteatum* shows a behaviour similar to that of *L. dufourii* [63] and far from that shown by more water-resistant species such as *L. albuferae* P.P. Ferrer et al. [63]. Furthermore, unlike most *Limonium* species sharing its habitat in the Valencian Community (*L. dufourii*, *L. girardianum* (Guss.) Kuntze, *L. virgatum* (Willd.) Fourr., *L. angustebracteatum* is a much more robust plant, bearing bigger and deeper root systems [11,28], which allows it to colonise dunes or other habitats unable for those species. These physiological and morphological features can explain that *L. angustebracteatum* was more abundant and less threatened than *L. dufourii*, listed as CR (Critically Endangered) under the IUCN classification (see [28,90]). However, even possessing this greater colonising capacity, *L. angustebracteatum* has vanished in some areas where it was abundant in the past, such as the Devesa de l’Albufera in Valencia (EL and PFG, pers. obs.), after abrupt alterations of the local ecosystem due to human activity (see [8,9]). Despite this local population decline, the results presented here allow us to propose *L. angustebracteatum* as a suitable species to be included in future ecological restoration projects because of its higher resilience compared to more delicate, endangered cohabitant taxa (i.e., *L. dufourii*).

## 4. Materials and Methods

### 4.1. Plant Material and Stress Treatments

One-year-old *L*. *angustebracteatum* plants, obtained from the germination of seeds, were used for this study. The plants were grown in a greenhouse with natural illumination, relative humidity of 65% and a 23–30 °C temperature range. Plants were individually placed in 12 cm diameter pots filled with a mixture of commercial peat and vermiculite (3:1) and watered regularly with tap water. Treatments were performed on 36 plants of uniform size, with six replicates per treatment. The following treatments were applied: control (irrigation with tap water, twice per week), water stress (complete withholding of irrigation) and salt stress (irrigation, twice per week, with aqueous solutions of 200, 400, 600 and 800 mM NaCl). After four weeks of treatment, the plants were cut and weighed, and their leaves were scanned to measure the leaf area. Part of the fresh material was frozen and partly dried in an oven at 65 °C for three days until a constant weight was recorded. The water content of the roots and leaves was measured according to the formula:

WC (%) = [(FW − DW)/FW] × 100(1)

### 4.2. Cryo-FESEM Preparations

Cryo-Field Emission Scanning Electron Microscopy (Cryo-FESEM) was performed at the Electronic Microscopy Service of the Polytechnic University of Valencia (QUORUM TECHNOLOGIES, Model PP3010T, Laughton, UK). Leaf samples were excised from adult plants grown under control conditions and physically fixed at ultra-low temperatures with slush nitrogen at −210 °C (cryogenisation); samples were maintained under low temperatures throughout the whole process in a preparation camera. Some samples were cracked with an inner stick to image transversal sections, and then a sublimation step (−90 °C for 10 min) was performed to eliminate residual liquid water. Finally, the samples were sputtered with platinum particles before imaging.

### 4.3. Light Microscopy

Sample preparation was carried out at the Microscopy Service of the Institute of Plant Molecular and Cell biology (IBMCP, Polytechnic University of Valencia). First, small sections of the leaves (4 mm^2^ approximately) were excised from plants grown under control, salt and drought conditions, collected on FAE (50% ethanol, 3.7% formaldehyde and 5% acetic acid) and subjected to vacuum for 15 min or until the samples were sunk. Then, the FAE solution was refreshed, and the samples were maintained at 4 °C. Next, the samples were dehydrated and included on paraffin with an automatic tissue processor (TP 1020, Leica, Germany). Briefly, the samples were incubated for 1 h with four increasing ethanol solutions (70, 90, 95, and 100%) and three commercial histoclear solutions. Then, inclusion was performed during two sequential incubations under a vacuum of 1 and 3 h with melted paraffin. Afterwards, samples were individually mounted in paraffin blocks and left to solidify at room temperature. Finally, the included samples were sliced (8 μm) with an RM2025 microtome (Leica Biosystems, Nussloch, Germany) and mounted on polysine-enriched slides.

For toluidine blue (TB) staining, sections on the slides were deparaffinised and rehydrated with sequential 10 min incubations with histoclear, ethanol 100%, 90% and 70%, and finally, with distilled water. Next, sections on the slides were incubated for 2 min with a 0.02% TB solution, rinsed with distillate water and dried. Finally, samples on the slides were mounted with distilled water and images were taken with a microscope Eclipse E1000 (Nikon, Tokyo, Japan).

### 4.4. Photosynthetic Pigments

Fresh leaves (50 mg) were used for the quantification of chlorophyll a (Chl a), chlorophyll b (Chl b) and carotenoids (Car) by the spectrophotometric method described by Lichtenthaler and Welburn [91]. Extraction was performed with one mL of ice-cold 80% acetone followed by overnight shaking at room temperature under dark conditions. Next, a centrifugation step was performed (13,300× *g*, 10 min at 4 °C). Finally, the absorbance of the supernatants (measured at 470, 646 and 663 nm) was used to estimate the concentrations of the pigments according to the equations previously described [91].

### 4.5. Quantification of Ions

Sodium (Na^+^), potassium (K^+^), chloride (Cl^−^) and calcium (Ca^2+^) levels in the roots and leaves were estimated according to Weimberg [92]. Samples (50 mg) of ground dry plant material were suspended in 15 mL of deionised water, heated at 95 °C in a water bath for one hour, followed by cooling on ice and filtration through a 0.45 µm nylon filter. The cations were quantified with a PFP7 flame photometer (Jenway Inc., Burlington, VT, USA) and the anion using a chlorimeter (Sherwood, model 926, Cambridge, UK).

### 4.6. Quantification of Osmolytes

Proline (Pro) was quantified according to the classical protocol by Bates et al. [93], with some modifications [94]. Pro was extracted from 50 mg of fresh leaves in 3% aqueous sulphosalicylic acid mixed with acid ninhydrin solution and incubated for one h at 95 °C. Next, the mixture was cooled on ice, and then two volumes of toluene were added. The absorbance of the supernatant was read at 520 nm, using toluene as a blank. Samples containing known Pro concentrations were assayed in parallel to obtain a standard curve. Pro concentration was expressed as μmol g^−1^ DW.

Glycine betaine (GB) was determined in 1-mL aqueous extracts prepared from 50 mg dry leaf material, according to published procedures [95,96]. The extract was supplemented with potassium iodide, kept on ice for 90 min and then extracted with 1,2-dichloroethane (pre-cooled at −20 °C). Finally, the absorbance of the sample was measured at 365 nm. GB content was expressed as µmol g^−1^ DW.

Total soluble sugars (TSS) were measured according to the method described by Dubois et al. [97] with some modifications [94]. First, fresh leaf material was ground in liquid N_2_ and extracted with 80% (*v*/*v*) methanol and mixed in a rocker shaker for 24 h. Next, samples were centrifuged at 13,300× *g* for 10 min, and supernatants were collected, diluted with water, and supplemented with concentrated sulphuric acid and 5% phenol. After 20 min incubation at room temperature, the absorbance was measured at 490 nm. TSS concentrations were expressed as equivalents of glucose, used as the standard (mg eq. glucose g^−1^ DW).

### 4.7. Statistical Analysis

Data were analysed using the program SPSS for Windows (SPSS Inc., Chicago, IL, USA). Before the analysis of variance, the Shapiro–Wilk test was used to check for the validity of the normality assumption and Levene’s test for the homogeneity of variance. If ANOVA requirements were accomplished, the significance of the differences between treatments was tested with a one-way ANOVA, followed by post hoc comparisons using Tukey’s HSD test at a significance level of *p* = 0.05. The mean values of all parameters measured in the plants were used for a principal component analysis (PCA). Hierarchical cluster analysis (HCA) and the corresponding heatmap were performed using the ClustVis 2.0 tool [98]. Rows were centred, and unit variance scaling was applied to rows. Both rows and columns were clustered using correlation distance and average linkage.

## 5. Conclusions

The results obtained revealed that *L. angustebracteatum* is a recretohalophyte highly resistant to salt stress. In addition to salt secretion through salt glands, its salt tolerance seems to depend on efficient osmotic adjustment by the foliar accumulation of high concentrations of ions (Na^+^ and Cl^−^, but also K^+^ and Ca^2+^) and the osmolytes proline and glycine betaine. The increase of K^+^ contents with increasing salinity also represents an especially remarkable tolerance mechanism as it can partially counteract the Na^+^ toxic effects. The contents of all four ions were significantly higher in the leaves than in the roots in non-stressed plants, indicating the presence of constitutive defence mechanisms based on active ion transport to the leaves, even at low external salinity. This constitutive response also included GB (but not Pro) accumulation since high absolute concentrations of the osmolyte were also measured in the leaves of the control plants.

On the contrary, the species is more susceptible to water deficit, but an active transport to the leaves of Na^+^, Cl^−^ and Ca^2+^ and a slight but significant increase in GB (but not Pro) contents were observed in water-stressed plants. These inorganic ions and the organic osmolyte contribute to osmotic balance under water stress conditions.

The large increase (over 40-fold) in K^+^ levels in roots of water-stressed plants supports the notion that K^+^ homeostasis plays a relevant role in the mechanisms of tolerance to both stressful conditions. Furthermore, Ca^2+^ can also be involved in salt and drought stress responses as an essential signalling molecule besides its osmotic effects.

## Figures and Tables

**Table 1:** Component weights in the PCA performed on *Limonium angustebracteatum* plants subjected to one month of stress treatments. Stronger correlations are shown in bold font. Abbreviations: LNo, number of leaves; LA, leaf area; RFW, root fresh weight; LFW, leaf fresh weight; RWC, root water content; LWC, leaf water content; Chl a, chlorophyll a; Chl b, chlorophyll b; Caro, carotenoids; Na^+^_r,_ sodium in roots; Na^+^_l_ sodium in leaves; Cl^−^_r_, chlorine in roots; Cl^−^_l_, chlorine in leaves; K^+^_r_, potassium in roots; K^+^_l_, potassium in leaves; Ca^2+^_r_, calcium in roots; Ca^2+^_l_, calcium in leaves; Pro, proline; GB, glycine betaine; TSS, total soluble sugars.

Trait	Component 1	Component 2	Component 3	Component 4
L No	**−0.285**	0.170	0.178	−0.059
LA	**−0.308**	0.006	0.267	−0.034
RFW	−0.212	**0.276**	−0.058	0.116
LFW	−0.156	**0.302**	−0.206	0.124
RWC	−0.090	**0.341**	0.184	0.033
LWC	−0.138	0.147	0.532	0.154
Chl a	0.001	0.053	−0.115	0.789
Chl b	**−0.262**	0.155	−0.304	0.104
Caro	0.017	**−0.268**	0.4445	0.133
Na^+^_r_	0.222	**0.267**	−0.019	−0.127
Na^+^_l_	**0.265**	0.194	−0.112	−0.205
Cl^−^_r_	0.224	**0.259**	0.121	−0.059
Cl^−^_l_	**0.304**	0.150	0.023	0.151
K^+^_r_	0.074	**−0.343**	−0.207	0.002
K^+^ _l_	**0.271**	−0.162	−0.088	0.314
Ca^2+^_r_	0.139	**0.328**	0.103	−0.114
Ca^2+^_l_	**0.263**	0.123	0.221	0.281
Pro	**0.279**	0.199	−0.083	0.037
GB	**0.328**	0.062	0.079	−0.104
TSS	0.198	−0.234	0.277	−0.019

## Data Availability

Data are contained within the article.

## References

[1] Erben M., Castroviejo S., Aedo C., Cirujano S., Laínz M., Montserrat P., Morales R., Muñoz Garmendia F., Navarro C., Paiva J., Soriano C. (1993). Limonium Mill. Flora Iberica.

[2] Sáez L., Rosselló J.A. (1999). Is *Limonium cavanillesii* Erben (*Plumbaginaceae*) really an extant species?. An. Jard. Bot. Madr..

[3] Crespo M.B. (2009). A new coastal species of *Limonium* (*Plumbaginaceae*) from Southeastern Spain. Folia Geobot..

[4] Ferrer-Gallego P.P., Navarro A., Pérez P., Roselló R., Rosselló J.A., Rosato M., Laguna E. (2015). A new polyploid species of *Limonium* (*Plumbaginaceae*) from the Western Mediterranean basin. Phytotaxa.

[5] Ferrer-Gallego P.P., Roselló R., Rosato M., Rosselló J.A., Laguna E. (2016). *Limonium albuferae* (*Plumbaginaceae*), a new polyploidy species from the Eastern Iberian Peninsula. Phytotaxa.

[6] Laguna E., Fos S., Ferrando-Pardo I., Ferrer-Gallego P.P., Grigore M.N. (2020). Endangered halophytes and their conservation lessons from Eastern Spain. Handbook of Halophytes: From Molecules to Ecosystems towards Biosaline Agriculture.

[7] Erben M. (1978). Die Gattung *Limonium* im Südwestmediterranien Raum. Mitt. Bot. Staatssamml. München.

[8] Yuan F., Leng B., Wang B. (2016). Progress in studying salt secretion from the salt glands in recretohalophytes: How do plants secrete salt?. Front. Plant Sci..

[9] Shabala S., Bose Y., Hedrich R. (2014). Salt bladders: Do they matter?. Trends Plant Sci..

[10] Caperta A.D., Róis A.S., Teixeira G., Garcia-Caparros P., Flowers T.J. (2020). Secretory structures in plants: Lessons from the Plumbaginaceae on their origin, evolution and roles in stress tolerance. Plant Cell Environ..

[11] Yuan F., Lyum M.J., Leng B.Y., Zhu X.G., Wang B.S. (2016). The transcriptome of NaCl-treated *Limonium bicolor* leaves reveals the genes controlling salt secretion of salt gland. Plant Mol. Biol..

[12] Zhang M., Chen Z., Yuan F., Wang B., Chen M. (2022). Integrative transcriptome and proteome analyses provide deep insights into the molecular mechanism of salt tolerance in *Limonium bicolor*. Plant Mol. Biol..

[13] Wyn Jones R., Storey R., Leigh R.A., Ahmad N., Pollard A., Marre E., Ciferri O. (1977). A hypothesis on cytoplasmic osmoregulation. Regulation of Cell Membrane Activities in Plants.

[14] Yeo A.R. (1983). Salinity resistance: Physiologies and prices. Physiol. Plant..

[15] Glenn E.P., Brown J.J., Blumwald E. (1999). Salt tolerance and crop potential of halophytes. Critic. Rev. Plant Sci..

[16] Flowers T.J., Munns R., Colmer T.D. (2014). Sodium chloride toxicity and the cellular basis of salt tolerance in halophytes. Ann. Bot..

[17] Flowers T.J., Colmer T.D. (2008). Salinity tolerance in halophytes. New. Phytol..

[18] Slama I., Abdelly C., Bouchereau A., Flowers T., Savouré A. (2015). Diversity, distribution and roles of osmoprotective compounds accumulated in halophytes under abiotic stress. Ann. Bot..

[19] Hasewaga P., Bressan R.A., Zhu J.K., Bohnert J. (2000). Plant cellular and molecular responses to high salinity. Annu. Rev. Plant Physiol. Plant Mol. Biol..

[20] Alvarez M., Savoure A., Szabados L. (2022). Proline metabolism as regulatory hub. Trends Plant Sci..

[21] Xu X., Feng J., Lü S., Lohrey G.T., An H., Zhou Y., Jenks M.A. (2014). Leaf cuticular lipids on the Shandong and Yukon ecotypes of saltwater cress, *Eutrema salsugineum*, and their response to water deficiency and impact on cuticle permeability. Physiol. Plant..

[22] Inan G., Zhang Q., Li P., Wang Z., Cao Z., Zhang H., Zhang C., Quist T.M., Goodwin S.M., Zhu J. (2004). Salt cress: A halophyte and cryophyte *Arabidopsis* relative model system and its applicability to molecular genetic analyses of growth and development of extremophiles. Plant Physiol..

[23] Sanchez D.H., Siahpoosh M.R., Roessner U., Udvardi M.K., Kopka J. (2008). Plant metabolomics reveals conserved and divergent metabolic responses to salinity. Physiol. Plant..

[24] Rahman M.M., Mostofa M.G., Keya S.S., Siddiqui M.N., Ansary M.M.U., Das A.K., Rahman M.A., Tran L.S.-P. (2021). Adaptive mechanisms of halophytes and their potential in improving salinity tolerance in plants. Int. J. Mol. Sci..

[25] Guevara D.R., Champigny M.J., Tattersall A., Dedrick J., Wong C.E., Li Y., Labbe A., Ping C.L., Wang Y., Nuin P. (2012). Transcriptomic and metabolomic analysis of Yukon *Thellungiella* plants grown in cabinets and their natural habitat show phenotypic plasticity. BMC Plant Biol..

[26] Kazachkova Y., Eshel G., Pantha P., Cheeseman J.M., Dassanayake M., Barak S. (2018). Halophytism: What have we learnt from *Arabidopsis thaliana* relative model systems?. Plant Physiol..

[27] Amtmann A. (2009). Learning from evolution: *Thellungiella* generates new knowledge on essential and critical components of abiotic stress tolerance in plants. Mol. Plant.

[28] Sévin D.C., Stählin J.N., Pollak G.R., Kuehne A., Sauer U. (2016). Global metabolic responses to salt stress in fifteen species. PLoS ONE.

[29] Liu X., Grieve C. (2009). Accumulation of chiro-inositol and other non-structural carbohydrates in *Limonium* species in response to saline irrigation waters. J. Am. Soc. Hortic. Sci..

[30] Laguna E. (1998). Flora Endémica, Rara o Amenazada de la Comunidad Valenciana.

[31] IUCN (2012). The IUCN Red List Categories and Criteria, Version 3.1.

[32] Laguna E. (2003). Priority Habitats of the Valencian Community.

[33] Fabregat C., Ranz J. (2015). Manual de Identificación de los Hábitats Protegidos de la Comunitat Valenciana (Decreto 70/2009).

[34] Feng Z., Sun Q., Deng Y., Sun S., Zhang J., Wang B. (2014). Study on pathway and characteristics of ion secretion of salt glands of *Limonium bicolor*. Acta Physiol. Plant..

[35] Santos J., Al-Azzawi M., Aronson J., Flowers T.J. (2016). eHALOPH a database of salt-tolerant plants: Helping put halophytes to work. Plant Cell Physiol..

[36] Flowers T.J., Galal H.K., Bromham L. (2010). Evolution of halophytes: Multiple origins of salt tolerance in land plants. Funct. Plant Biol..

[37] Dassanayake M., Larkin J.C. (2017). Making plants break a sweat: The structure, function, and evolution of plant salt glands. Front. Plant Sci..

[38] Grigore M.N., Toma C., Grigore M.N. (2021). Morphological and Anatomical Adaptations of Halophytes: A Review. Handbook of Halophytes: From Molecules to Ecosystems towards Biosaline Agriculture.

[39] Lu C., Yuan F., Guo J., Han G., Wang C., Chen M., Wang B. (2021). Current understanding of role of vesicular transport in salt secretion by salt glands in recretohalophytes. Int. J. Mol. Sci..

[40] Leng B.Y., Yuan F., Dong X.X., Wang J., Wang B.S. (2018). Distribution pattern and salt excretion rate of salt glands in two recretohalophyte species of *Limonium* (*Plumbaginaceae*). S. Afr. J. Bot..

[41] Li J., Yuan F., Liu Y., Zhao Y., Wang B., Chen M. (2020). Exogenous melatonin enhances salt secretion from salt glands by upregulating the expression of ion transporter and vesicle transport genes in *Limonium bicolor*. BMC Plant Biol..

[42] Gao Y., Zhao B., Jiao X., Chen M., Wang B., Yuan F. (2021). Coupled development of salt glands, stomata, and pavement cells in *Limonium bicolor*. Front. Plant Sci..

[43] Mi P., Yuan F., Guo J., Han G., Wang B. (2021). Salt glands play a pivotal role in the salt resistance of four recretohalophyte *Limonium* Mill. species. Plant Biol..

[44] Xu X., Zhou Y., Mi P., Wang B., Yuan F. (2021). Salt-tolerance screening in *Limonium sinuatum* varieties with different flower colors. Sci. Rep..

[45] Ni X.-L., Tan L.-L., Shen X.-D. (2012). Developmental and anatomical studies of the salt gland in *Limonium aureum*. Acta Bot. Boreal. Occid. Sin..

[46] de Fraine E. (1916). The morphology and anatomy of the genus *Statice*, as represented at Blake ney point. I. *Statice binervosa*, G.E Smith and *Statice bellidifolia* D.C. (= *S. reticulata*). Ann. Bot..

[47] Colombo P. (2002). Morpho-anatomical and taxonomical remarks on *Limonium* (*Plumbaginaceae*) in Sicily. Flora Medit..

[48] Ding F., Chen M., Sui N., Wang B.S. (2010). Ca^2+^ significantly enhanced development and salt-secretion rate of salt glands of *Limonium bicolor* under NaCl treatment. S. Afr. J. Bot..

[49] Ding F. (2013). Effects of salinity and nitric oxide donor sodium nitroprusside (SNP) on development and salt secretion of salt glands of *Limonium bicolor*. Acta Physiol. Plant..

[50] Parida A.K., Veerabathini S.K., Kumari A., Agarwal P.K. (2016). Physiological, anatomical and metabolic implications of salt tolerance in the halophyte *Salvadora persica* under hydroponic culture condition. Front. Plant Sci..

[51] Al Hassan M., Gohari G., Boscaiu M., Vicente O., Grigore M.N. (2015). Anatomical modifications in two *Juncus* species under salt stress conditions. Not. Bot. Horti Agrobo..

[52] Munns R., Tester M. (2008). Mechanisms of salinity tolerance. Annu. Rev. Plant Biol..

[53] Flowers T.J., Hajibagheri M.A., Clipson N.J.W. (1986). Halophytes. Q. Rev. Biol..

[54] González-Orenga S., Grigore M.-N., Boscaiu M., Vicente O. (2021). Constitutive and induced salt tolerance mechanisms and potential uses of *Limonium* Mill. species. Agronomy.

[55] Morales M.A., Olmos E., Torrecillas A., Sánchez-Blanco M.J., Alarcon J.J. (2001). Differences in water relations, leaf ion accumulation and excretion rates between cultivated and wild species of *Limonium* sp. grown in conditions of saline stress. Flora.

[56] Ding F., Song J., Ruan Y., Wang B.S. (2009). Comparison of the effects of NaCl and KCl at the roots on seedling growth, cell death and the size, frequency and secretion rate of salt glands in leaves of *Limonium sinense*. Acta Physiol. Plant..

[57] Xianzhao L., Chunzhi W., Qing S. (2013). Screening for salt tolerance in eight halophyte species from Yellow River Delta at the two initial growth stages. ISRN Agron..

[58] Souid A., Gabriele M., Longo V., Pucci L., Bellani L., Smaoui A., Abdelly C., Hamed K. (2016). Salt tolerance of the halophyte *Limonium delicatulum* is more associated with antioxidant enzyme activities than phenolic compounds. Funct. Plant Biol..

[59] Hameed A., Gulzar S., Aziz I., Hussain T., Gul B., Khan M.A. (2015). Effects of salinity and ascorbic acid on growth, water status and antioxidant system in a perennial halophyte. AoB Plants.

[60] Al Hassan M., Pacurar A., López-Gresa M.P., Donat-Torres M.P., Llinares J.V., Boscaiu M., Vicente O. (2016). Effects of salt stress on three ecologically distinct *Plantago* species. PLoS ONE.

[61] Grieve C.M., Poss J.A., Grattam S.R., Sheuse P.J., Lieth J.H., Zeng L. (2005). Productivity and mineral nutrition of *Limonium* species irrigated with saline wastewaters. Hort. Sci..

[62] González-Orenga S., Ferrer-Gallego P.P., Laguna E., López-Gresa M.P., Donat-Torres M.P., Verdeguer M., Vicente O., Boscaiu M. (2019). Insights on salt tolerance of two endemic *Limonium* species from Spain. Metabolites.

[63] González-Orenga S., Donat-Torres M., Llinares J.V., Navarro A., Collado F., Ferrer-Gallego P., Laguna E., Vicente O., Boscaiu M. (2021). Multidisciplinary studies supporting conservation programmes of two rare, endangered *Limonium* species from Spain. Plant Soil.

[64] Al Hassan M., Estrelles E., Soriano P., López-Gresa M.P., Bellés J.M., Boscaiu M., Vicente O. (2017). Unraveling salt tolerance mechanisms in halophytes: A comparative study on four Mediterranean *Limonium* species with different geographic distribution patterns. Front. Plant Sci..

[65] González-Orenga S., Llinares J.V., Al Hassan M., Fita A., Collado F., Lisón P., Vicente O., Boscaiu M. (2020). Physiological and morphological characterisation of *Limonium* species in their natural habitats: Insights into their abiotic stress responses. Plant Soil.

[66] González-Orenga S., Al Hassan M., Llinares J.V., Lisón P., López-Gresa M.P., Verdeguer M., Vicente O., Boscaiu M. (2019). Qualitative and quantitative differences in osmolytes accumulation and antioxidant activities in response to water deficit in four Mediterranean *Limonium* species. Plants.

[67] Tabot P.T., Adams J.B. (2014). Salt secretion, proline accumulation and increased branching confer tolerance to drought and salinity in the endemic halophyte *Limonium linifolium*. S. Afr. J. Bot..

[68] Flowers T.J., Yeo A.R., Baker D.A., Hall J.L. (1988). Ion relation of salt tolerance. Solute Transport in Plant Cells and Tissues.

[69] Alarcón J.J., Morales M.A., Torrecillas A., Sánchez-Blanco M.J. (1999). Growth, water relations and accumulation of organic and inorganic solute in the halophytes *Limonium latifolium* cv. Avignon and its interspecific hybrid *Limonium caspia* × *Limonium latifolium* cv. Bettlaard during salt stress. J. Plant Physiol..

[70] Zia S., Egan T.P., Khan M.A. (2008). Growth and selective ion transport of *Limonium stocksii* Plumbaginacea under saline conditions. Pak. J. Bot..

[71] Flowers T.J., Troke P., Yeo A.R. (1977). Mechanism of salt tolerance in halophytes. Annu. Rev. Plant Physiol..

[72] Greenway H., Munns R. (1980). Mechanisms of salt tolerance in non-halophytes. Annu. Rev. Plant Physiol..

[73] Marschner P. (2012). Marschner’s Mineral Nutrition of Higher Plants.

[74] Wang M., Zheng Q., Shen Q., Guo S. (2013). The critical role of potassium in plant stress response. Int. J. Mol. Sci..

[75] Al Hassan M., Chaura J., López-Gresa M.P., Borsai O., Daniso E., Donat-Torres M.P., Mayoral O., Vicente O., Boscaiu M. (2016). Native-invasive plants vs. halophytes in Mediterranean salt marshes: Stress tolerance mechanisms in two related species. Front. Plant Sci..

[76] Feng Z.T., Deng Y.Q., Zhang S.C., Liang X., Yuan F., Hao J.L., Zhang J.C., Sun S.F., Wang B.S. (2015). K(+) accumulation in the cytoplasm and nucleus of the salt gland cells of *Limonium bicolor* accompanies increased rates of salt secretion under NaCl treatment using NanoSIMS. Plant Sci..

[77] Parida A.K., Das A.B. (2005). Salt tolerance and salinity effects on plants: A review. Ecotoxicol. Environ. Saf..

[78] Manishankar P., Wang N., Köster P., Alatar A.A., Kudla J. (2018). Calcium signaling during salt stress and in the regulation of ion homeostasis. J. Exp. Bot..

[79] Seifikalhor M., Aliniaeifard S., Shomali A., Azad N., Hassani B., Lastochkina O., Li T. (2019). Calcium signaling and salt tolerance are diversely entwined in plants. Plant Signal. Behav..

[80] Carter C.T., Grieve C.M., Poss J.A. (2005). Salinity effects on emergence, survival, and ion accumulation of *Limonium perezii*. J. Plant Nutr..

[81] Aliniaeifard S., Shomali A., Seifikalhor M., Lastochkina O., Hasanuzzaman M., Tanveer M. (2020). Calcium signaling in plants under drought. Salt and Drought Stress Tolerance in Plants. Signaling and Communication in Plants.

[82] Gagneul D., Aïnouche A., Duhazé C., Lugan R., Larher F.R., Bouchereau A. (2007). A reassessment of the function of the so-called compatible solutes in the halophytic Plumbaginaceae *Limonium latifolium*. Plant Physiol..

[83] Rhodes D., Hanson A.D. (1993). Quaternary ammonium and tertiary sulfonium compounds in higher plants. Annu. Rev. Plant Physiol. Plant Mol. Biol..

[84] Giri J. (2011). Glycinebetaine and abiotic stress tolerance in plants. Plant Signal. Behav..

[85] Rhodes D., Nadolska-Orczyk A., Rich P., Läuchli A., Lüttge U. (2002). Salinity, osmolytes and compatible solutes. Salinity: Environment-Plants-Molecules.

[86] Khan M.A., Ungar I.A., Showalter A.M. (2000). The effect of salinity on the growth, water status, and ion content of a leaf succulent perennial halophyte, *Suaeda fruticosa* (L.) Forssk. J. Arid Environ..

[87] Tipirdamaz R., Gagneul D., Duhazé C., Aïnouche A., Monnier C., Özkum D., Larher F. (2006). Clustering of halophytes from an inland salt marsh in Turkey according to their ability to accumulate sodium and nitrogenous osmolytes. Environ. Exp. Bot..

[88] Furtana G.B., Duman H., Tipirdamaz R. (2013). Seasonal changes of inorganic and organic osmolyte content in three endemic *Limonium* species of Lake Tuz (Turkey). Turk. J. Bot..

[89] Gil R., Boscaiu M., Lull C., Bautista I., Lid N.A., Vicente O. (2013). Are soluble carbohydrates ecologically relevant for salt tolerance in halophytes?. Funct. Plant Biol..

[90] Aguilella A., Fos S., Laguna E. (2010). Catálogo Valenciano de Especies de Flora Amenazada.

[91] Lichtenthaler H.K., Wellburn A.R. (1983). Determinations of total carotenoids and chlorophylls a and b of leaf extracts in different solvents. Biochem. Soc. Trans..

[92] Weimberg R. (1987). Solute adjustments in leaves of two species of wheat at two different stages of growth in response to salinity. Physiol. Plant..

[93] Bates L.S., Waldren R.P., Teare I.D. (1973). Rapid determination of free proline for water-stress studies. Plant Soil.

[94] González-Orenga S., Leandro M.E.D.A., Tortajada L., Grigore M.N., Llorens A.A., Ferrer-Gallego P.P., Laguna E., Boscaiu M., Vicente O. (2021). Comparative studies on the stress responses of two *Bupleurum* (*Apiaceae*) species in support of conservation programmes. Environ. Exp. Bot..

[95] Grieve C.M., Grattan S.R. (1983). Rapid assay for determination of water soluble quaternary ammonium compounds. Plant Soil.

[96] Nawaz K., Ashraf M. (2010). Exogenous application of glycine betaine modulates activities of antioxidants in maize plants subjected to salt stress. J. Agron. Crop Sci..

[97] Dubois M., Gilles K.A., Hamilton J.K., Reberd P.A., Smith F. (1956). Colorimetric method for determination of sugars and related substances. Anal. Chem..

[98] Metsalu T., Vilo J. (2015). ClustVis: A web tool for visualizing clustering of multivariate data using Principal Component Analysis and heatmap. Nucleic Acids Res..

